# Analysing the hidden curriculum: use of a cultural web

**DOI:** 10.1111/medu.12072

**Published:** 2013-02

**Authors:** Liz Mossop, Reg Dennick, Richard Hammond, Iain Robbé

**Affiliations:** 1 School of Veterinary Medicine and Science, University of NottinghamNottingham, UK; 2 Medical Education Unit, Queen's Medical Centre, The University of NottinghamNottingham, UK; 3 Institute of Primary Care & Public Health, Cardiff University School of MedicineCardiff, UK

## Abstract

**CONTEXT** Major influences on learning about medical professionalism come from the hidden curriculum. These influences can contribute positively or negatively towards the professional enculturation of clinical students. The fact that there is no validated method for identifying the components of the hidden curriculum poses problems for educators considering professionalism. The aim of this study was to analyse whether a cultural web, adapted from a business context, might assist in the identification of elements of the hidden curriculum at a UK veterinary school.

**METHODS** A qualitative approach was used. Seven focus groups consisting of three staff groups and four student groups were organised. Questioning was framed using the cultural web, which is a model used by business owners to assess their environment and consider how it affects their employees and customers. The focus group discussions were recorded, transcribed and analysed thematically using a combination of *a priori* and emergent themes.

**RESULTS** The cultural web identified elements of the hidden curriculum for both students and staff. These included: core assumptions; routines; rituals; control systems; organisational factors; power structures, and symbols. Discussions occurred about how and where these issues may affect students’ professional identity development.

**CONCLUSIONS** The cultural web framework functioned well to help participants identify elements of the hidden curriculum. These aspects aligned broadly with previously described factors such as role models and institutional slang. The influence of these issues on a student’s development of a professional identity requires discussion amongst faculty staff, and could be used to develop learning opportunities for students. The framework is promising for the analysis of the hidden curriculum and could be developed as an instrument for implementation in other clinical teaching environments.

## Introduction

Despite the complexities of defining medical professionalism, the topic’s importance is not disputed and the teaching and assessment of professionalism represent areas of focus for many educational institutions.^1,2^ Medical schools developing or reviewing curricula are placing emphasis on professionalism teaching equivalent to that placed on existing, more traditional components. Similar changes are also occurring in other clinical training programmes, including in dentistry,^3^ pharmacy^4^ and veterinary medicine.^5^ Although each discipline has distinct elements, there are many areas of common ground.

The teaching of professionalism is challenging, not least because of the difficulties of defining the attributes to be included in this complex skill set.^2,6^ There is much discussion as to how and when this teaching should occur and by whom it should be delivered.^7^ An area of focus for many refers to the difficulty of teaching the ‘right thing to do’ in an environment in which students are exposed to a wide spectrum of behaviour and attitudes, both good and bad. This environment has several contexts, including that of the relatively uncontrollable workplace. No matter how well taught professionalism is, it is possible for the influence of the culture and environment – the hidden curriculum – to undermine the work of well-intentioned teachers.

### The hidden curriculum

The hidden curriculum is classically defined as ‘a set of influences that function at the level of organisational structure and culture’,^8^ which manipulate teachers and learners in the context of both the formal and informal curricula. During clinical training, students undergo a process of professional identity formation^9^ as they learn the ‘rules’ of the new community of practice they are joining.^10^ This process of situated learning, widely recognised as a key element of learning professionalism,^11,12^ is heavily influenced by the often non-overt attitudes and behaviours of peers and teachers. There is the potential for conflict between the process of becoming a good professional and that of learning what is necessary to ‘fit’ into a new environment.

Theoretical discussions around the nature of professionalism or how it should be taught have therefore been criticised when the influence of the hidden curriculum is ignored.^8,13–16^ Students need to learn what it truly means to be a doctor and medical education leaders must be aware of the issues in the hidden curriculum influencing this process.^17,18^ It has been suggested that it may be necessary to attempt to change the hidden curriculum if negative aspects are identified.^19,20^ Faculty development is often included as a key component of this process of change that represents an attempt to influence staff to model more ‘appropriate’ behaviours.^21^

### Components of the hidden curriculum

**Figure 1:**
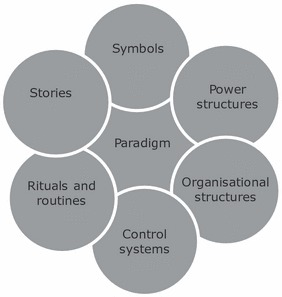
The cultural web^22^

Hidden influences include elements such as role models, rules and regulations, the use of institutional slang and resource allocation.^8^ Similar components are included in descriptions of the culture of businesses. Business analysts use cultural models as a means of recognising customer perceptions and develop strategy accordingly. One particular model, the cultural web (Fig. 1),^22^ identifies several different themes that can be used to consider the mission and values of a company. This model is used to organise information from employees and customers in order to provide an overview of strategy. The applicability of these elements in the educational context can easily be recognised.

### Identifying the hidden curriculum

Although the elements of the hidden curriculum and its importance as a concept are often discussed, this dimension of the curriculum is rarely formally assessed despite the need to establish whether remediation may be necessary. Hafferty^8^ discusses four domains for examination (policy development, evaluation, resource allocation and institutional slang), but how this examination should be carried out is not detailed.

Specific attempts have been made to analyse the hidden curriculum within institutions, but without an established methodology for doing so, this is difficult.^23,24^ The validated ‘C3 Instrument’ identifies the patient-centredness of a school’s hidden curriculum, but does not analyse beyond this context.^25^ Students appear to be good at recognising influences around them during their studies, and several studies have analysed their narratives or interviews.^26–28^ Typical factors to emerge include role models, specific events and informal learning. However, students may not be completely reliable in their ability to identify these influences as their understanding of professionalism may be variable.^29^This is perhaps not surprising given the general lack of consensus on the definition of medical professionalism,^6,13^ although one study does suggest that students can successfully use faculty professionalism narratives to identify influences over their learning.^30^

Ethnographic studies give the most valid picture of ‘unseen’ influences,^31^ but these are costly and time-consuming to implement. A combination of quantitative and qualitative methodologies that systematically collect data from multiple stakeholders is probably the most effective strategy, but this may be unfeasible for many institutions and, again, has not been validated sufficiently.^19^

The hidden curriculum reaches far beyond the lecture theatre and, particularly in clinical education, encompasses numerous environments and contexts. It is not generally included in standard course evaluations, despite the implications of its influence on teaching and learning. This may be because analysis is not straightforward. The overall aim of this study therefore was to attempt to use a cultural web, adapted from the business context, as a framework for identifying elements of the hidden curriculum in one veterinary medicine school in the UK. The analysis placed particular focus on the elements that influence the development of professional identity in order to assess the influence of the hidden curriculum on this process. The study then considered how the school should manage these elements and assessed the usefulness of this methodology.

## Methods

The institution under study is a new veterinary medicine and science school in the UK, which graduated its first cohort of 95 students in 2011. The school’s 5-year degree programme in veterinary medicine follows a clinically integrated spiral curriculum that provides early clinical experience and integrated professional skills teaching. The final year of study is delivered through a community-based model, using university-employed faculty staff to teach in regional veterinary practices covering the full range of species and specialties.

A qualitative approach using focus groups was chosen in order to establish perceptions of staff and students within the school. A total of seven groups were led by an independent researcher (staff groups) and a trained student researcher (student groups). It was hoped that the use of a student researcher and the consequent absence of an authority figure would help the student groups feel more able to contribute. Selection for the groups was carried out using a combination of stratified random and purposive sampling, and participation was voluntary. Each contributor gave individual consent and his or her anonymity was assured.

Johnson *et al.*’s cultural web^22^ was developed as a framework by which data could be collected and analysed. The participants were introduced to the themes in the web and the facilitators used a semi-structured approach to discuss the non-overt influences within the curriculum in each category. Discussion around the potential for these factors to manipulate the development of behaviours in students was encouraged.

Focus group discussions were recorded and transcribed. Thematic analysis of the data was undertaken using a combination of *a priori* themes derived from the model developed by Johnson *et al.*^22^ and emergent themes. NVivo Version 8.0 (QSR International Pty Ltd, Doncaster, Vic, Australia) was used to manage this process. Initial analysis was undertaken by two researchers until agreement was reached on the first two transcripts. Analysis was completed by a single researcher once the coding had been confirmed. An iterative process was used to draw out themes of interest and relevance to the area under study. These themes were then cross-checked for similarities and differences, and grouped together accordingly across the *a priori* themes.

This study received ethical approval from the institution’s ethical review panel.

## Results

The sampling strategy employed ensured the three staff focus groups consisted of a cross-section of the staff population by age, gender and role within the school. Both academic and support staff were represented. The student groups included a cross-section of students according to age, year of study and gender.

### Thematic analysis

**Figure 2:**
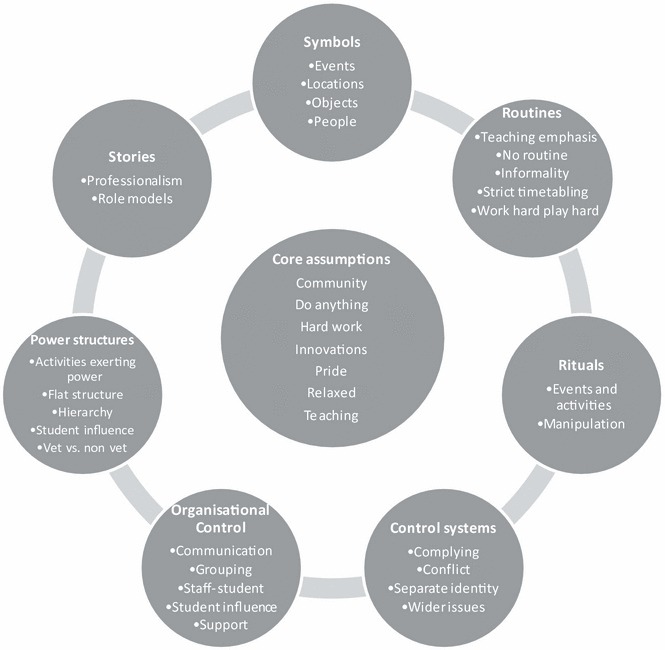
Issues contributing to the hidden curriculum identified using the cultural web^22^ as a model

The cultural model appeared to successfully shape data collection and analysis; several distinct themes emerged within the *a priori* codes and are represented diagrammatically in Fig. 2.

### Core assumptions

Participants in all groups were quick to identify the core beliefs and strategies of the school. Several themes in relation to this emerged, with particular reference to the feeling of community within the school. Staff and students identified a team-like atmosphere:

‘I think it’s a very supportive place to work. Everybody seems to be working together as a team to achieve the same goals.’

(Staff group 2)

‘We’re like a little school, it’s like school still…’

(Student group 3)

‘…it [is] like a family atmosphere. Everybody seems to be friendly and very welcoming.’

(Student group 4)

Participants judged their environment as innovative and experimental, as might be expected in a new school; risk taking was discussed as an element of this:

‘The reason I came here was because it was taking risks and because it had a kind of a forward-thinking approach and it was kind of pushing the boundaries slightly…’

(Student group 1)

This discussion about the school as an innovative community seemed to encourage participants to identify differences between it and other environments they had experienced, reinforcing the community theme:

‘…it’s modern, it’s new, it’s progressive and I think in that sense it distinguishes itself from other academic departments…’

(Student group 4)

The staff groups in particular identified the existence of a ‘do anything’ attitude within the school, in which individuals might ignore traditional boundaries of roles and responsibilities:

‘...there weren’t many people to start with, you did end up doing, I don’t know, a bit of finance, a bit of placements, a bit of everything, [...] to start with it was very much all hands on deck, and one day you might be carrying a fibreglass horse across the car park and the next you could be doing anything…’

(Staff group 3)

Participants agreed that there was an awareness within the school of the need to work hard. Students discussed the rigours of a full timetable and examination schedule, and staff commented on the workloads of both their own roles and students.

A sense of pride emerged in discussions about what the school had achieved, and with reference to being a member of an identified community. The feeling of belonging to the school was very important and staff were able to recognise this within the student body:

‘They’re very proud of their Vet School and that kind of thing. And I think some of that comes from being new, the new Vet School […] that wish to go to AVS [Association of Veterinary Students] or wherever else and be as good as everyone else…’

(Staff group 2)

The school’s community atmosphere was discussed further within this theme. The school was described as being friendly and relaxed, within both the staff and student bodies, as well as in relations between them:

‘It is a very friendly place to work, and quite senior professors and stuff are very approachable.’

(Staff group 2)

Faculty staff participants expressed the belief that a core paradigm referred to a focus on, and respect for, teaching, sometimes to the detriment of research activities:

‘My teaching skills are appreciated within the Vet School, and that a commitment to teaching is seen as a valid commitment to professionalism within the organisation.’

(Staff group 2)

### Routines

Participants were asked to discuss routines they identified as part of the day-to-day activities of the school and as reflecting the way things are done. Somewhat ironically, an emerging theme concerned the lack of routine within the school, with the exception of timetabling, which was perceived by students as extremely strict:

‘Certainly like the whole teaching, learning and assessment has felt a bit lastminute.com a lot of the time…’

(Student group 1)

An informal approach in the routine of the school was felt to prevail on occasion and represented something students struggled to manage:

‘And it’s a bit difficult where, at the start, some members of staff will say, “Call me by my first name.” And then when you talk to someone else about a member of staff about them, they’re like, “Oh, you should call me Doctor so-and-so or Professor so-and-so.” And it’s kind of – it would be nicer to have a sort of, across the whole school say, everyone will be called by their first name. Apart from in correspondence. Or everyone will be called by their full title kind of thing. Something just a bit more structured than kind of it flip-flopping every five minutes.’

(Student group 1)

Demonstrating a degree of overlap between routines and core assumptions, the school’s emphasis on teaching activities was highlighted by some participants as reflecting its routines. Discussed within most groups was the routine of ‘work hard, play hard’, although students felt this was not a simple paradigm:

‘I think it’s kind of work or play hard but there’s kind of a lot of unspoken rules about it as well.’

(Student group 1)

### Rituals

The groups were asked to describe what they felt constituted a ritual within the school with reference to an event or activity with meaning that had influenced themselves or others. The annual dean’s cocktail party, a social event for staff and students, was highlighted as an important social ritual. Other events, such as the annual student cabaret, the student mentoring programme, and teaching-related activities such as tutorials and visits to farms in the school, branded ‘Land Rovers’, were also discussed as important school rituals.

There was some discussion in the student groups about students who took part in rituals such as the annual student cabaret, and how this affected staff:

‘The Vet Review’s a really important ritual. Because it’s [a] really nice way to end sort of things. And it sort of shows the lecturers that, because obviously there are some people that are always going to be in it, and some people that will probably never be in it. And I think it’s a way of saying, actually it’s quite a nice way to say back to those lecturers that have been in it, actually we may have just like basically destroyed your character, but actually we thank you for making that school a bit more of a laugh than it could have been.’

(Student group 3)

There was also dialogue around socialising generally between students and staff, and how this represented an important ritual.

### Control systems

The concept of control of the school, through external sources, was discussed; an emergent theme concerned a mixture of compliance and conflict, whereby the school is able to act independently of the central university, but still must comply with certain regulations, or does comply for different reasons:

‘The faculty do tend to support us, because we are different and obviously they’re different as well. So you have to have different things. And then they will take it to the university, but more often than not, the university will overrule and say no, you’ve got to do it this way, that sort of thing. But I think it sort of just comes down, fed down through the faculty and then back to the school.’

(Staff group 3)

Students also made this distinction:

‘Even though [...] it doesn’t feel as though you’re part of the University of Nottingham at times, that was what I was basing my decision to choose this course [on]. In that I knew that the University of Nottingham wouldn’t be willing to risk their reputation to have a vet course that failed.’

(Student group 1)

Awareness of the existence of a separate identity also falls into the theme of control, discussions of which overlapped with those around conflict and compliance. Members of the school generally felt that the school was very distinct from the central university, both geographically and psychologically:

‘But I doubt that a lot of main campus would really know like who we were, and like hadn’t really heard of our campus really. Because obviously we don’t really go there for, well us as a vet school don’t go there for lectures.’

(Student group 3)

The staff groups also discussed wider issues of control beyond that of the university as an institution. Some felt that the current political situation would result in the encroaching of control from other sources, such as students who are empowered by their payment of higher fees and whose expectations are consequently changed.

### Organisational structure

Discussion around the structure of the school from within centred on several themes. Communication within the school was perceived very differently. Some felt that communication was generally good, whereas others were aware of changes associated with the growth of the school, and issues with rumours emerging within the student body:

‘Not knowing anything, having rumours from loads of sides, and you know rumours develop within student populations rapidly and just escalating things and different rotations, different people hearing different things…’

(Staff group 2)

‘And I think they should – they can improve by deciding things between themselves first before telling you. And then just telling you properly.’

(Student group 1)

The issue of social groupings arose as a theme in different contexts. Staff discussed student integration between years, and referred to the emergence of groups that perhaps did not encourage communication and mixing. Students discussed groups in the context of ‘cliques’:

‘...at vet school there are aspects which are quite cliquey almost, you know. And if like you’re not in that group then, you know, you don’t get involved kind of thing.’

(Student group 3)

Also within the theme of routines was a prominent issue referring to how staff and students interact within the school, and how this was interpreted, particularly with reference to social events:

‘But I think, really, things like the Vet Review, like the student–staff sports day, which really shape the kind of community of people. There is not one big distance between staff and the students if you want to play football against each other or do bicycle racing. So I think this is really an important part of student–staff relations.’

(Staff group 2)

‘It makes them seem more human really. [...] It’s nice to kind of have that barrier broken down so you can talk to them on a more like friend basis…’

(Student group 1)

However, the subject of this interaction elicited some questions and storytelling from students and it was apparent that some students struggled to identify social barriers and boundaries in certain situations.

The ability of students to exert influence and control the school was discussed. This overlaps with the theme of ‘power’ and will be described in the next section.

Support available within the school arose as a topic in discussion about organisation. Staff felt students were able to access a huge amount of support to help them complete a demanding course, and students equally valued this aspect of the culture and did not feel afraid to access it:

‘Sometimes you can think, “Well, yes. Actually if I had a problem, I’d know who to go to.” And that would be fine.’

(Student group 1)

### Power structures

The theme of power was discussed extensively in the groups. Opinions on who was in control of the school and how others reacted to this varied. There was dialogue regarding the presence or lack of a hierarchy within both staff and students. The management structure of the school was described as relatively ‘flat’, which added to feelings of friendliness and openness:

‘It feels like you can go into anybody’s office and say something and you’ll be listened to, probably, much more than in some other places I’ve worked where it feels like there’s very much a hierarchy, where there are people that you can’t approach.’

(Staff group 2)

However, some participants did not find this easy to manage:

‘It’s not obvious who [are] line managers, though, for people. You tend to go, who is – because it’s such a flat structure, you’re never quite sure of okay, who is line manager there? Who do I go to?’

(Staff group 1)

Students also differed in their perceptions of the school hierarchy. They focused on the staff–student hierarchy and how this affected behaviour:

‘And it’s very difficult when there’s different levels [...]. But there are situations like that where it’s [a] very difficult line for what’s acceptable as a student what’s unacceptable for a member of staff. Whether they should even – whether they’d have to live by the same rules, I’m not sure. But it certainly does vary.’

(Student group 1)

There was discussion within the staff focus groups of certain activities that exerted influence over others, such as when an individual’s action resulted in greater perceived power and higher status. Student influence emerged within both the power and organisational control themes. Both staff and students commented on the power of the student body and its ability to influence decisions within the school:

‘To a large extent the students dictate the way things are done within the school. So even if you do put a serious point across, it then goes back to certain committees where students will have a lot of input into it and what was a serious sometimes just gets dressed – brushed under the carpet and you know, forgotten about, really.’

(Staff group 1)

One of the striking similarities among the groups concerned the distinction between ‘vets and non-vets’, which was often raised within the general conversation. Vets were often described as being in a more powerful position in both the school’s and students’ eyes. The dichotomy between the two groups was clearly perceived by many and students also described the importance of being labelled a ‘vet student’:

‘... [students are] very keen to find out if you’re a vet or not…’

(Staff group 2)

‘...it is a bit of like a status thing to say, “Yes. I’m a vet student”…’

(Student group 1)

### Stories

In general, storytelling occurred throughout the group discussions, but some distinct themes emerged within these stories, often relating to role-modelling and its influence on students’ professionalism:

‘I think perhaps a good example is swearing by members of staff. Some will do it quite freely in lectures. And there’s not a problem there. But other lecturers get very upset by it. And then it’s the point of if the staff member acts like that, does that allow the students to swear? Or doesn’t it? And there’s a very difficult line.’

(Student group 1)

### Symbols

Symbols broadly fell into four categories pertaining to events, locations, people and objects, respectively. Branded clothing, purchased from the school, was highlighted as something symbolic to many students. The dean’s dog was described as a symbolic ‘person’, representing the enthusiastic nature of the staff in general.

## Discussion

The cultural web helped to reveal the perceptions of participants regarding the components of the hidden curriculum, and shaped data collection and simplified analysis. Discussions were very broad and inevitably included some aspects of evaluation of teaching and learning from participants. However, using the framework themes makes it possible to draw out aspects of the curriculum that specifically influence professional identity formation. These influences include a feeling of community and innovation, but also examples of rebellion and occasional conflict, all of which will potentially shape the learning environment. Examples of role models were also clearly identified by students. They were able to use negative role models as triggers for reflection on what was right and wrong in a manner that helped them to shape their own identity.

### Interpreting the hidden curriculum

The emergent themes should be interpreted in the context of developing professionals in order to assess how these issues influence this process and debate whether this environment needs to be managed.

A sense of community is likely to help students during their studies. Students may struggle to ‘fit in’ and achieve legitimate peripheral participation initially,^10^ but once they have established how things work, developing a feeling of belonging is very important and should help their learning of professionalism as they assimilate the behaviour role-modelled by those around them.

However, the opposite might also be concluded because the security of the school environment may contrast sharply with the workplace environments students will experience upon graduation. In a profession in which levels of stress and dropout rates are high,^32,33^ the gap between the strongly supportive institutional community and the potentially less supportive practice environment is huge. Will this experience of engaging in a community help or hinder graduates as they enter more isolated workplaces as professionals? This may represent an element of learning that needs to be provided or reinforced by the formal professionalism curriculum.

The occasional superior identity of clinicians referred to in the narratives may encourage graduates entering the workplace as professionals to feel superior to their patients and co-workers. The school may be viewed as encouraging this aspect of professionalism without reinforcing the other side of the social contract.^34^ Professionals must take care not to overly exert their authority and to ensure that they contribute effectively to society.^35^ This is a strong message which should be reinforced by the hidden curriculum.

By contrast, the legitimisation of independent thought may have a positive influence on students as they need to develop into autonomous professionals. An entrepreneurial spirit may benefit those who enter private practice. However, sanctioning actions independently of a controlling body such as the university may be interpreted as approving rebellion. The constant conflict between professional autonomy and organisational control will be a theme in students’ future professional lives as they develop the ability to negotiate the complexities of the workplace^36^ and thus the experience of a similar type of conflict may be seen as good preparation for the future.

### Managing the hidden curriculum

Once influences have been identified, they may need to be managed; any changes that are necessary should occur at individual, faculty and institutional levels.^19,32^ In this institution, a new formal professionalism curriculum has been proposed, partly in response to the findings of this study. This curriculum harnesses students’ abilities to recognise role models by using critical event analysis, a form of guided reflection to help students identify good and bad professional attitudes and behaviours.^37^ Faculty development will be key to delivering this kind of teaching.^38^

### The use of the cultural web

The final objective of this study was to assess the validity of this methodology and discuss whether it might be used in some format by other clinical education institutions. The cultural web appears to have enabled the identification of previously recognised elements of the hidden curriculum, plus several additional areas. For example, policy development, evaluation and assessment, resource allocation and the use of institutional slang are described by Hafferty^8^ and all emerged in the current study. The cultural web would therefore appear to have functioned well as a framework, helping to map both data collection and analysis.

### Strengths and limitations of the study

The qualitative nature of this study means that issues have been explored in depth, although the results will have limited generalisability to other institutions. Focus groups provide an excellent source of qualitative data, but by their nature are influenced by group dynamics and require the input of an experienced facilitator.^39^ Further triangulation of data would help to increase the reliability of the findings and potentially assist in the ongoing development of a professional culture assessment tool with which to monitor the clinical education environment.

## Conclusions

The hidden curriculum is important to consider within any institution in which developing professionals are taught. Some interesting aspects of the present school’s hidden curriculum were drawn out with the current method of analysis, providing faculty staff with useful discussion points as the environment evolves. The power of role-modelling should be harnessed to provide reflective learning experiences for students.

This study has demonstrated the usefulness of the cultural web as a framework for performing this analysis. The framework may therefore be valuable for other institutions, and could be used to develop a more convenient survey, or as a basis for student discussions.

### Contributors

LM designed the study and led the data analysis and write-up of the paper. RD and RH contributed to the study design and to the critical revision of the paper. IR contributed to the design of the study, collected the data, and contributed to data analysis and the redrafting and critical revision of the paper. All four authors approved the final manuscript for submission.

### Acknowledgements

The authors are grateful to all contributors to this study.

### Funding

Funded by The University of Nottingham Lord Dearing Fellowship Award.

### Conflicts of interest

none.

### Ethical approval

ethical approval was sought and obtained from the institutional ethics review committee at the School of Veterinary Medicine and Science, University of Nottingham.
